# The anti‐ageing hormone klotho induces Nrf2‐mediated antioxidant defences in human aortic smooth muscle cells

**DOI:** 10.1111/jcmm.12996

**Published:** 2016-10-03

**Authors:** Giuseppe Maltese, Paraskevi‐Maria Psefteli, Benedetta Rizzo, Salil Srivastava, Luigi Gnudi, Giovanni E. Mann, Richard C.M. Siow

**Affiliations:** ^1^ Cardiovascular DivisionBritish Heart Foundation Centre of Research ExcellenceFaculty of Life Sciences & MedicineKing's College LondonLondonUK; ^2^ Department for Life Quality StudiesAlma Mater StudiorumUniversity of BolognaBolognaItaly

**Keywords:** Klotho, Nrf2, vascular smooth muscle, angiotensin II, atherosclerosis, haeme oxygenase‐1, peroxiredoxin‐1, glutathione, antioxidant, ageing

## Abstract

Vascular ageing in conditions such as atherosclerosis, diabetes and chronic kidney disease, is associated with the activation of the renin angiotensin system (RAS) and diminished expression of antioxidant defences mediated by the transcription factor nuclear factor erythroid 2‐related factor 2 (Nrf2). The anti‐ageing hormone klotho promotes longevity and protects against cardiovascular and renal diseases. Klotho has been shown to activate Nrf2 and attenuate oxidative damage in neuronal cells, however, the mechanisms by which it protects against vascular smooth muscle cell VSMC dysfunction elicited by Angiotensin II (AngII) remain to be elucidated. AngII contributes to vascular ageing and atherogenesis by enhancing VSMC oxidative stress, senescence and apoptosis. This study demonstrates that soluble klotho (1 nM, 24 hrs) significantly induces expression of Nrf2 and the antioxidant enzymes haeme oxygenase (HO‐1) and peroxiredoxin‐1 (Prx‐1) and enhances glutathione levels in human aortic smooth muscle cells (HASMC). Silencing of Nrf2 attenuated the induction of HO‐1 and Prx‐1 expression by soluble klotho. Furthermore, soluble klotho protected against AngII‐mediated HASMC apoptosis and senescence *via* activation of Nrf2. Thus, our findings highlight a novel Nrf2‐mediated mechanism underlying the protective actions of soluble klotho in HAMSC. Targeting klotho may thus represent a therapeutic strategy against VSMC dysfunction and cardiovascular ageing.

## Introduction

Age‐related disorders including diabetes, hypertension and chronic kidney disease are associated with activation of renin angiotensin system (RAS) and increased risk of vascular disease 1, 2, 3. AngII plays a central role in the development of hypertension and atherosclerosis through an increase in reactive oxygen species (ROS) generation and down‐regulation of endogenous antioxidant defence systems such as Nrf2 4, 5, 6. AngII has been also shown to induce senescence and apoptosis in vascular smooth muscle cells (VSMC) *via* activation of the cell cycle regulation proteins p53 and p21 7. The redox‐sensitive transcription factor Nrf2 mediates endogenous antioxidant protection against oxidative stress associated with cardiovascular pathologies 8. In response to a range of inducers, Nrf2 translocates into the nucleus binding to the antioxidant response elements (ARE) in the promoter region of target antioxidant defence genes such as haeme oxygenase‐1 (HO‐1), peroxiredoxin‐1 (Prx‐1) and enzymes involved in reduced glutathione (GSH) synthesis 9.

Klotho is a renal protein originally reported as a regulator of the ageing process in mice 10, 11, 12. Klotho deficiency is associated with decreased lifespan and accelerated vascular ageing, whereas its overexpression has been shown to confer vascular protection through reduction in oxidative stress and arterial calcification 10. Klotho is not only predominantly expressed in the kidney as a membrane ‐protein but also exists as a circulating soluble form resulting from a proteolytic cleavage 13.

Soluble klotho acts as a hormone that confers antioxidant, anti‐senescence and anti‐apoptotic effects in endothelial and renal cells 14, 15. Nrf2 activation has been identified as a mechanism by which klotho enhances antioxidant defences in neuronal and epithelial cells 16, 17, however, to date the involvement of Nrf2 in klotho‐mediated vascular cell protection has not been investigated.

This study has examined the role of Nrf2 in the protective effects of soluble klotho against AngII‐induced oxidative stress, apoptosis and senescence in human aortic smooth muscle cells (HASMC). We provide novel mechanistic evidence that soluble klotho induces the antioxidant defence enzymes HO‐1 and Prx‐1, enhances Nrf2 expression and levels of reduced GSH, and attenuates AngII‐mediated apoptosis and senescence *via* activation of Nrf2.

## Material and methods

### Culture of HASMSs

Human aortic smooth muscle cells were purchased from Lonza Group Ltd. and cultured in DMEM (Sigma‐Aldrich, UK) supplemented with 10% (v/v) foetal calf serum, 1% L‐glutamine, penicillin (100 U/ml) and streptomycin (100 μg/ml). Experiments were performed in HASMC between passages 6 and 12. Cells were treated with either AngII (200 nΜ) or vehicle (DMSO 0.01%, 0–72 hrs), or recombinant human klotho (0–1 nM, 0–72 hrs) R&D systems, Abingdon, UK.

### Immunoblotting

Cells were lysed with an SDS buffer (2% w/v SDS, 10% v/v glycerol, 50 mM Tris–HCl, pH 6.8) containing protease inhibitor cocktail. Total protein content was determined with the bicinchoninic acid assay (Pierce, ThermoFisher Scientific, Northumberland, UK). Denatured samples were separated by SDS‐PAGE, transferred to a polyvinylidine difluoride (Merck Millipore, Watford, UK) membrane and probed with the following primary antibodies: HO‐1 (BD Transduction Laboratories, Oxford, UK), Prx‐1 (Gift from Prof. Tetsuro Ishii, University of Tsukuba, Japan), Nrf2 (Santa Cruz Biotechnology, Dallas, TX, USA) or α‐tubulin (Millipore) as a reference protein. Enhanced chemiluminescence was used to visualize bands on the membrane which were quantified by densitometric analysis.

### Nrf2 knock‐down

Human aortic smooth muscle cells were transfected 24 hrs after seeding in 24‐well plates with 40 pmol/well Nrf2‐specific small interfering RNA (siRNA) or scrambled siRNA (Santa Cruz Biotechnology) using DharmaFECT 4 transfection reagent (GE Healthcare Life Science, Amersham, UK), as previously described 18.

### Measurement of intracellular‐reduced GSH

A fluorometric assay was used to measure reduced GSH levels as previously described 18. Human aortic smooth muscle cells were washed with PBS (4°C) before lysis using 6.5% trichloroacetic acid for 10 min. on ice. Samples were incubated with phosphate (80 mM), ethylenediaminetetraacetic acid (5 mM) buffer (pH 8) containing o‐phthalaldehyde (0.1% w/v) in methanol for 25 min. Fluorescence intensity (ex: 350 nm, em: 420 nm) was measured with a microplate reader (Chameleon V; Hidex, Turku, Finland) and normalized to protein content using the BCA assay.

### Generation of superoxide

Superoxide generation was determined using L‐012 chemiluminescence (Wako Chemical Industries, Osaka, Japan), as previously described 18, 19. Human aortic smooth muscle cells were treated with recombinant human klotho (1 nM) for 24 hrs in the absence or presence of AngII (200 nM) for the final 4‐hr period and then incubated with Krebs buffer containing L‐012 (20 nM) at 37°C, in the continued presence of klotho (1 nM) and AngII (200 nM) and in the presence or absence of superoxide dismutase (SOD; 100 U/ml). Luminescence was monitored over a 10‐min. period at 37°C using a microplate luminometer (Chameleon V; Hidex) and mean light units measured over 1 sec. normalized to cellular protein content.

### Assessment of apoptosis

Annexin V binding to phosphatidylserine can be used as a marker of early apoptotic events 20. Binding of Cy5‐conjugated annexin V to HASMC was assessed using a commercial kit (Biotium‐bioscience, Fremont, CA, USA). Cells were co‐stained with Hoechst 33342 (Sigma‐Aldrich) to identify nuclei and visualized using a fluorescence microscope (Nikon, Gotenba, Japan) and images were acquired using a cooled CCD camera (Hamamatsu Photonics, Hamamatsu, Japan). Equivalent number of cells were captured for each field and fluorescence intensity was determined using analysis software (Image J; NIH, Bethesda, MD, USA).

### Assessment of senescence

Senescent HASMC were identified by detection of β‐galactosidase (β‐gal) activity, a biomarker of cellular senescence 21, at pH 6.0 using a senescence‐associated β‐gal assay kit (Cell Signalling Technology, Danvers, MA, USA). Briefly, cultures were washed with PBS and fixed for 10–15 min. at room temperature. After two further washes with PBS the cells were incubated with the β‐gal staining solution (1 mg/ml 5‐bromo‐4‐chloro‐3‐indonyl‐βD‐galactopyranoside solution, at pH 6.0 for 12 hrs at 37°C). Cells were visualized with a microscope (TMS; Nikon) and blue stained senescent cells identified. At least three representative fields of view were captured for each treatment condition using a digital camera (Canon, Tokyo, Japan).

### Statistical analysis

Data denote mean ± S.E.M. of measurements in at least 3–5 independent HASMC cultures. The effects of treatment on protein expression and GSH levels were evaluated with an unpaired Student's *t*‐test. Comparisons of two or more variables in the same experiment were conducted using either a one‐ or two‐way anova with the Tukey or Sidak multiple comparisons test respectively.

## Results

### Soluble klotho induces HO‐1 and Prx‐1 expression in a Nrf2‐dependent manner and enhances GSH levels

**Figure 1:**
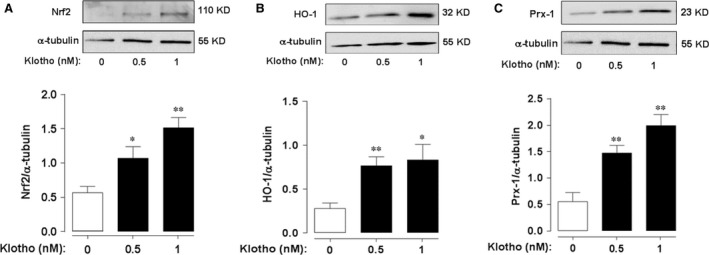
Klotho induces expression of Nrf2, HO‐1 and Prx‐1 in HASMC. Cells were treated with human recombinant soluble klotho (0–1 nM, 24 hrs). Representative immunoblots and densitometric analysis of Nrf2 (**A**), HO‐1 (**B**) and Prx‐1 (**C**) protein expression relative to α‐tubulin. Data denote mean ± S.E.M., *n* = 4–5, *P < 0.05, **P < 0.01 relative to untreated cells.

**Figure 2:**
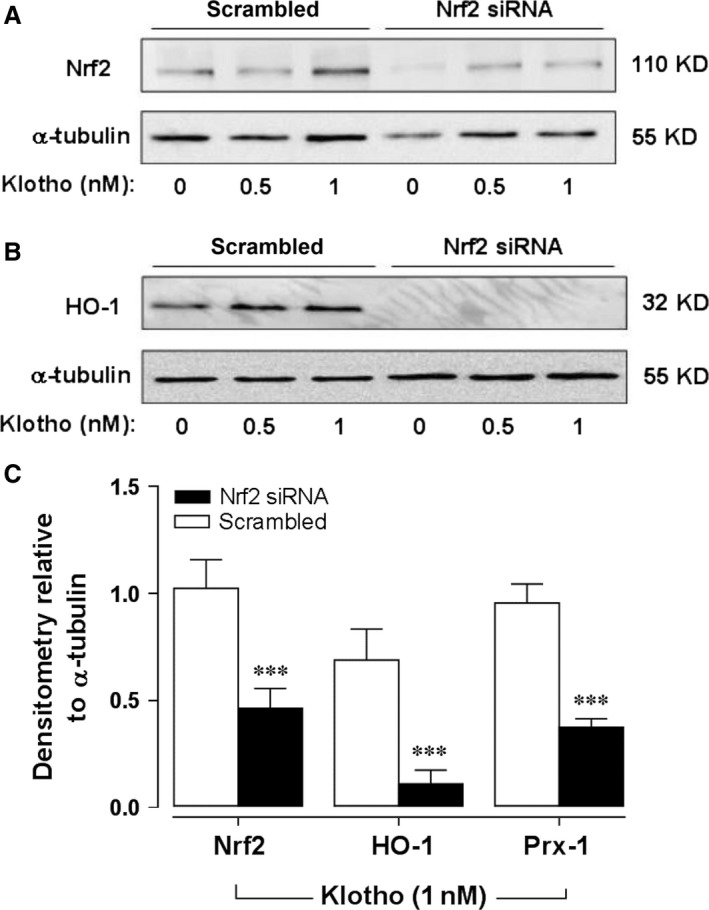
Nrf2‐mediated induction of HO‐1 and Prx‐1 in HASMC. Cells were transfected with scrambled or Nrf2 siRNA prior to treatment with soluble klotho (1 nM, 24 hrs) and Nrf2 (**A**) and HO‐1 (**B**) expression determined ay immunoblot analyses. (**C**) Densitometric analysis of Nrf2, HO‐1 and Prx‐1 protein expression relative to α‐tubulin following klotho treatment (1 nM, 24 hrs) in control (open bars) or Nrf2‐deficient cells after siRNA knock‐down (solid bars). Data denote mean ± S.E.M., *n* = 3, ***P < 0.001.

To assess whether klotho enhances antioxidant defences in HAMSC, we examined the effects of recombinant human soluble klotho on the expression of the Nrf2‐regulated enzymes HO‐1 and Prx‐1. We initially demonstrated that soluble klotho enhances expression of Nrf2 in a dose‐dependent manner (Fig. 1A). As shown in Figure 1B and C, treatment of HASMC with recombinant human soluble klotho (0.5 or 1.0 nM) for 24 hrs significantly increased the expression of both HO‐1 and Prx‐1. Knock‐down of Nrf2 using siRNA significantly attenuated the enhanced Nrf2, HO‐1 and Prx‐1 expression in response to soluble klotho treatment (Fig. 2).

We next sought to characterize the role of klotho in regulating levels of reduced GSH, the major intracellular thiol antioxidant 22. HASMC were treated with soluble klotho (0.1 or 0.5 nM, 24 hrs) and intracellular levels of GSH were measured by fluorescence. Soluble klotho enhanced GSH levels in a dose‐dependent manner (GSH nmol/mg protein – Control: 22.7 ± 2.0, 0.1 nM klotho: 36.7 ± 3.5*; 0.5 nM klotho: 41.73 ± 3.916*, *P < 0.05, *n* = 3).

### Klotho reduces AngII‐mediated superoxide generation

**Figure 3:**
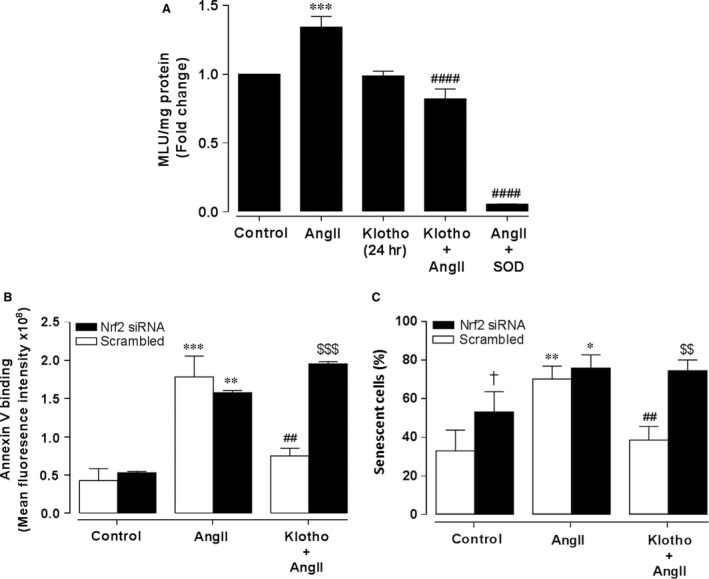
Soluble klotho attenuates AngII‐mediated superoxide generation, apoptosis and senescence in HASMC. Superoxide generation (**A**) was measured by L‐012 chemiluminescence and mean light units (MLU) measured in live cells after incubation with soluble klotho (1 nM, 24 hrs) in the absence or presence of angiotensin II (AngII, 200 nM) for the final 4 hrs. Some cells were treated with superoxide dismutase (SOD, 200 U/ml) during the measurement period. Data denote mean ± S.E.M., *n* = 4, ***P < 0.001 relative to control cells, ####*P* < 0.0001 relative to cells treated with AngII. Apoptosis (**B**) was assessed by annexin V binding in cells treated with AngII (200 nM, 18 hrs) in the presence or absence of soluble klotho pre‐treatment (1 nM, 24 hrs). Graph shows quantification of annexin V fluorescence following Nrf2 siRNA knock‐down (solid bars) or transfection with scrambled siRNA (open bars) prior to treatments with soluble klotho. Data denote mean ± S.E.M., *n* = 3, **P < 0.01, ***P < 0.001 relative to untreated cells, ##*P* < 0.01 relative to cells transfected with scrambled siRNA prior to AngII treatment, $$$*P* < 0.001 relative to respective cells transfected with scrambled siRNA. Senescence (**C**) was assessed using the senescence‐associated β‐galactosidase (SA‐β‐gal) activity assay. Following transfection with scrambled or Nrf2 siRNA, cells were pre‐incubated with soluble klotho (1 nM, 24 hrs) prior to treatment with AngII (200 nM, 72 hrs) in the continued presence of soluble klotho. Cells were stained for SA‐β‐gal activity and the number of senescent cells quantified by image analyses as a percentage of total cell number in at least 3 fields of view. Data denote mean ± S.E.M., *n* = 3, *P < 0.05, **P < 0.01 relative to respective untreated cells, ##*P* < 0.01 relative to scrambled siRNA transfected cells treated with AngII alone, $$*P* < 0.01 relative to control cells transfected with scrambled siRNA, †*P* < 0.05 relative to scrambled siRNA‐transfected control cells.

To further investigate the actions of klotho in protecting against oxidative stress in HASMC, we evaluated its effect on AngII‐mediated superoxide generation. Cells were treated with soluble klotho (1 nM) for 24 hrs and AngII (200 nM) or vehicle were added for the last 4 hrs in the continued presence of soluble klotho. Superoxide generation was enhanced by AngII treatment (Fig. 3A), while pre‐treatment with klotho or addition of SOD (100 U/ml) during the assay significantly attenuated the AngII‐mediated superoxide generation.

### Effects of soluble klotho on AngII‐mediated apoptosis

As apoptosis is a crucial event in atherosclerosis 23, we assessed the effects of soluble klotho against AngII‐mediated apoptosis in HASMC. Cells were treated with AngII (200 nM) for 18 hrs and an increased number of apoptotic cells was detected by annexin V fluorescence (Fig. 3B). Treatment of cells with soluble klotho (1 nM, 24 hrs), prior to AngII (200 nM, 18 hrs), significantly reduced the number of apoptotic cells. Knock‐down of Nrf2 using siRNA attenuated klotho‐mediated protection against apoptosis, which suggests that up‐regulation of Nrf2 signalling represents an underlying mechanism by which soluble klotho confers protection against Ang II‐mediated apoptosis.

### Klotho reduces Ang II‐mediated senescence

As AngII induces senescence of VSMC and accelerates the development of atherosclerosis 7, we investigated whether soluble klotho prevents AngII‐mediated HASMC senescence. HASMC were treated with AngII (200 nM, 72 hrs) to induce senescence, assessed by SA‐β‐gal staining. As shown in Figure 3C, AngII increased the number of SA‐β‐gal‐positive senescent cells and pre‐treatment with soluble klotho (1 nM, 24 hrs) significantly decreased the number of senescent cells. Knock‐down of Nrf2 using siRNA abolished the protective effects of klotho pre‐treatment against AngII‐induced HASMC senescence.

## Discussion

Activation of RAS and down‐regulation of Nrf2 signalling and antioxidant gene expression are associated with the development of age‐related vascular diseases 24. In this study, we provide the first evidence that the soluble form of the anti‐ageing protein, klotho, activates Nrf2 in HASMC, resulting in an enhanced expression of the antioxidant enzymes HO‐1 and Prx‐1 which are regulated by ARE in their promoter regions. In addition, GSH levels were significantly increased following klotho treatment, likely arising from the induction of Nrf2‐related genes involved in the synthesis of GSH 25. Our findings also demonstrate that klotho attenuates AngII‐induced apoptosis and senescence *via* activation of the Nrf2 signalling pathway.

AngII is known to be an inducer of ROS production in VSMC which contributes to age‐related vascular inflammation and atherogenesis 26. NADPH oxidases (Nox) and the mitochondrial respiratory chain represent the major molecular sources of ROS generation in response to AngII in VSMC 27, 28, which can express multiple Nox isoforms, including Nox1, Nox2, Nox4 and Nox5 29. Although Nox2 is found in all vascular cells, it is expressed to a lesser extent in arterial VSMC, whereas Nox4 is likely to be the main isoform expressed in human aortic VSMC 30.

Enhanced expression of HO‐1 and Prx‐1 has been shown to protect against atherosclerosis in murine models 9, 31, 32. We have previously reported that Nrf2 mediates an important protective response to oxidative stress through the expression of HO‐1 and Prx‐1 in human and murine VSMC 33, 34, 35. In animal models of vascular disease, activators of Nrf2 signalling can restore cellular redox homoeostasis by increasing the expression of antioxidant enzymes 9.

A reciprocal regulation between klotho and Nrf2 signalling has been shown in studies using klotho mutant mice 36, 37. Klotho deficiency *in vivo* has been reported to be associated with decreased hepatic cytoplasmic and nuclear levels of Nrf2, conversely klotho overexpression results in an increase in nuclear Nrf2 and activation of the ARE in the promoter of antioxidant genes 36. Furthermore, down‐regulation of klotho in airway epithelial cells has been shown to contribute to the activation of oxidative and inflammatory pathways and is associated with diminished Nrf2 signalling 38. We demonstrate that soluble klotho is an inducer of Nrf2 in HASMC in a dose‐dependent manner. Treatment of HASMC with soluble klotho prior to AngII exposure significantly attenuated ROS generation, which is consistent with the existing literature on the protective effects of klotho in the vasculature. Although Six *et al*. reported an acute increase in ROS generation by soluble klotho in HASMC 39, klotho gene delivery in rat aortic VSMC results in protection against AngII‐induced superoxide production *via* down‐regulation of Nox2 protein expression mediated by the cAMP‐PKA pathway 40. In cardiomyocytes from rodents with chronic kidney disease, soluble klotho has been reported to inhibit ROS generation through the attenuation of Nox2 and Nox4 expression 41. A reduction in renal mitochondrial oxidative stress and DNA damage has been shown in klotho transgenic mice 42. Taken together, the reduction in ROS generation observed in our study following klotho pre‐treatment is likely to have arisen from diminished AngII‐induced Nox and mitochondrial ROS generation as well as increased scavenging of ROS mediated by the enhanced expression of Nrf2‐regulated antioxidant enzymes.

We have provided the first demonstration that Nrf2‐mediates the increase in expression of HO‐1 and Prx‐1 in VSMC following treatment with soluble klotho. This is likely to have occurred through nuclear localization of Nrf2 and binding to the ARE in the promoter regions of Nrf2‐responsive genes 9. Although the effect of AngII on Nrf2 signalling was not investigated in this study, AngII has been shown to elicit nuclear accumulation of Nrf2 in aortic VSMC from normotensive but not spontaneously hypertensive rats, as an antioxidant defence response to enhanced ROS generation and oxidative stress 6.

In addition to oxidative stress, VSMC apoptosis contributes to atherogenesis 43. The present study demonstrates that soluble klotho protects HASMC from AngII‐induced apoptosis. The anti‐apoptotic and antioxidant effects of klotho have been previously shown in cultured endothelial cells and reported to be linked with the p53/p21 and mitogen‐activated protein kinase pathways 44. Renin angiotensin system activation is a characteristic event in the progression of cardiovascular disease and is associated with klotho deficiency 45. Conversely, administration of exogenous soluble klotho may offer cardiovascular‐renal protection in chronic kidney disease by blunting the activation of RAS 45. A bidirectional link between klotho and RAS has been described and confirmed by previous work from our group 46, 47.

Smooth muscle cell senescence is associated with the development of vascular disease 48. We are the first to show that soluble klotho attenuates AngII‐induced senescence in HASMC mediated by Nrf2 as knock‐down of Nrf2 attenuated the protective effect of klotho. Protection against hydrogen peroxide‐mediated senescence by klotho has been previously reported in human fibroblasts and umbilical vein endothelial cells but the role of Nrf2 was not assessed 49, 50, 51.

This study represents a novel report on the antioxidant, anti‐apoptotic and anti‐senescence effects of soluble klotho in HASMC mediated by an up‐regulation of Nrf2 signalling and induction of antioxidant enzymes. Nrf2 has a role in vasoprotection and it is known to be regulated by intracellular redox status 52. It is likely that protein kinases may regulate the activation of Nrf2 following treatment with soluble klotho 37. The activation of GSK3‐β diminishes antioxidant gene expression by enhancing nuclear exclusion and inactivation of Nrf2 53. Klotho has been identified as inhibitor of GSK3‐β in renal epithelial cells 54, thus suppression of GSK3‐β signalling by klotho represents a possible mechanism by which it enhances Nrf2‐mediated antioxidant enzyme expression and confers protection against AngII in HASMC. Additional studies are necessary to further elucidate the cellular signalling pathways that link soluble klotho with Nrf2 signalling.

In conclusion, our findings provide novel mechanistic insights into the vasculoprotective effects of soluble klotho mediated by the Nrf2 antioxidant signalling pathway, and emphasize the therapeutic potential of targeting klotho to activate Nrf2 in cardiovascular and other ageing‐related diseases.

## Conflicts of interest

The authors confirm that there are no conflicts of interest.
